# Dendritic Cells Decreased the Concomitant Expanded Tregs and Tregs Related IL-35 in Cytokine-Induced Killer Cells and Increased Their Cytotoxicity against Leukemia Cells

**DOI:** 10.1371/journal.pone.0093591

**Published:** 2014-04-04

**Authors:** Ying Pan, Qianshan Tao, Huiping Wang, Shudao Xiong, Rui Zhang, Tianping Chen, Lili Tao, Zhimin Zhai

**Affiliations:** Department of Hematology, the Second Hospital of Anhui Medical University, and Hematology Research Center, Hefei, Anhui, People's Republic of China; Penn State University, United States of America

## Abstract

Regulatory T cells (Tregs) are potent immunosuppressive cells and essential for inducing immune tolerance. Recent studies have reported that Tregs and Tregs related cytokines can inhibit the antitumor activity of cytokine-induced killer (CIK) cells, but dendritic cells co-cultured CIK (DC-CIK) cells can be used for induction of a specific immune response by blocking of Tregs and TGF-β, IL-10. As a novel identified cytokine, IL-35 is specially produced by Tregs and plays an essential role in immune regulation. However, it remains unknown whether IL-35 roles in tumor immunotherapy mediated by CIK and DC-CIK cells. In this study, we cultured CIK and DC-CIK cells from the same healthy adult samples, and investigated their phenotype, proliferation, cytotoxic activity against leukemia cell lines K562 and NB4 by FCM and CCK-8, measured IL-35, TGF-β and IL-10 protein by ELISA, detected Foxp3, IL-35 and IL-35 receptor mRNA by Real-time PCR, respectively. We found Tregs and IL-35 concomitantly expanded by a time-dependent way during the generation of CIK cells, but DC significantly down-regulated the expression of them and simultaneously up-regulated the proliferation ability as well as cytotoxic activity of CIK cells against leukemia cell lines. Therefore, our data suggested that DC decreased concomitant expanded Tregs and Tregs related IL-35 in CIK cells and might contribute to improve their cytotoxicity against leukemia cells in vitro.

## Introduction

Cytokine-induced killer (CIK) cells are heterogeneous cell populations including a major effector cell population expressing both T cell marker CD3 and natural killer (NK) cell marker CD56, and display powerful cytotoxicity against tumor cells in a non-major histocompatibility (MHC) restricted manner [1]. As the primary candidate for adoptive cell immunotherapy, CIK cells have confirmed benefit and safety for many patients with hematological malignancies and solid tumors over the past two decades [2]–[4].

However, the main functional properties of CIK cells have still been limited by some inhibitory factors [5]. Regulatory T cells (Tregs) are potent immunosuppressive cells that promote tumor growth and invasion by inducing immune escape and suppressing anti-tumor immune response [6]–[8]. Some studies reported that Tregs also significantly decreased the cytotoxicity of CIK cells, and the classical inhibitory cytokines TGF-β as well as IL-10 might participate into the immune regulation processes of Tregs in CIK cells [9], [10]. So, it is believed that depletion or down-regulation of Tregs and Tregs related cytokines in CIK cells will enhance their killing activity [9], [10]. Fortunatlly, recent studies found that co-culturing CIK cells with dendritic cells (DC) could be used for induction of a specific immune response by blocking of Tregs as well as the cytokines TGF-β and IL-10 [11]–[13].

Furthermore, it is known to all that Tregs can mediate suppression through multiple mechanisms and molecules, especially through cytokine-dependent mechanisms [14], [15]. Except for the classical TGF-β and IL-10, IL-35 composed of IL-12 α subunit p35 and IL-27 β subunit Epstein-Barr virus-induced gene 3 (EBI3) is a newly identified immunosupressive cytokine [16], [17]. As a novel member of IL-12 cytokine family, IL-35 is specifically produced by Tregs and contribute to suppressing T cell proliferation and function [16]–[18]. Interestingly, the newest studies showed that IL-35, rather than TGF-β or IL-10, was required in Tregs-mediated maximal immune suppression [16], [19]. However, it is still unknown the expression of IL-35 in CIK cells and the role of DC in regulating Tregs-related IL-35 in CIK cellls.

In this study, we cultured CIK and DC-CIK cells from the same samples derived from healthy adults, then investigated the phenotype, proliferation and cytotoxicity against leukemia cells, respectively. Moreover, the expression of IL-35 and IL-35 receptor (IL-35R) were analyzed and compared to determine the IL-35 characteristics between CIK and DC-CIK cells.

## Materials and Methods

### Ethics Statement

All participants signed a statement of written informed consent. The procedures described in this study were approved by the ethics committee of Anhui Medical University.

### DC culture and identification *in vitro*

Seven healthy adults were approved by the institutional ethics committee and enrolled in the present study. The peripheral blood mononuclear cells (PBMC) were cultured with 10% fetal bovine of RPMI 1640 containing GM-CSF (500 U/ml),IL-4 (500 U/ml),replaced with half of flesh medium and supplement cytokines every 3 days,and added TNF-α (50 U/ml) on 72 h before harvest to induce DC mature. Then, the induced DC were indentified with FITC-CD83, PE-CD1a and APC-CD11c purchased from Beckman Coulter Immunotech (Miami, FL, USA) by flow cytometer FC-500 (Beckman Coulter, Miami, FL, USA).

### Generation of CIK and DC-CIK cells

PBMC from seven health adults were enriched by density gradient centrifugation. Then, PBMC were plated at a density of 5×10^6^ cells/ml in GT-T551 medium (Takara, Dalian, China) with the addition of 1000 U/ml IFN-γ (Peprotech, Rocky Hill, NJ, USA) on the first day. 24 hours later, 50 ng/ml anti-human CD3 monoclonal antibody (eBioscience, San Diego, CA, USA), 500 IE/ml IL-1α (Peprotech, Rocky Hill, NJ, USA) and 500 IE/ml IL-2 (Peprotech, Rocky Hill, NJ, USA) were added. The DC and CIK cells were co-cultured on day 7 at the ratio of 1∶5. Then, the cells were incubated at 37°C with 5% CO_2_ and subcultured every 3 days in fresh GT-T551 medium containing 500 IE/ml IL-2. Both CIK and DC-CIK cells were expanded in vitro over 28 days and partly harvested on day 7, 14, 21 and 28 for analysis, respectively.

### Phenotype analysis

The phenotype of both CIK and DC-CIK cells were incubated with different monoclonal antibodies specific for human antigens which purchased from Beckman Coulter Immunotech (Miami, FL, USA), except for APC-Foxp3 purchased from eBioscience (San Diego, CA, USA)), then analyzed by flow cytometer FC-500 (Beckman Coulter, Miami, FL, USA). The following monoclonal antibodies were used: FITC-CD25, PE-CD127 and PC5-CD4 for Tregs; FITC-CD4, PE-CD8 and PC5-CD3 for T cells; FITC-CD3 and PE-CD56 for NK or NKT cells. Moreover, in order to further investigate the expression of Tregs, the Foxp3 mRNA expression was also measured by Applied Biosystems 7500 Real-time Polymerase Chain Reaction (PCR) (Life, Grand Island, NY, USA). The primers (Table 1) were purchased from Takara Company (Takara, Dalian, China).

**Table 1:** Real-time PCR primers sequences.

Primers	Sense	Anti-sense
**p35 ** [20]	5′-GATGAGCTGATGCAGGCC	5′-AGTCCTCCACCTCGTTGTCCGTGA
**EBI3** [20]	5′-GCAGACGCCAACGTCCAC	5′-CCAGTCACTCAGTTCCCCGT
**IL-12Rβ2** [21]	5′-GTATGACCTTGTTTGTCTGCAAGC	5′-CTGTAAACGGTCTCAGATCTCGCA
**gp130** [21]	5′-TGTCACG-TTCACAGACGTGGTCCT	5′-CCAAGTTGAGGTATCTT-TGGTCCT
**Foxp3**	5′-CAGCTGCCTACAGTGCCCCTAG	5′-ATTTGCCAGCAGTGGGTAG
**GAPDH**	5′-CCACATCGCTCAGACACCAT	5′-CCAGGCGCCCAATACG

### Cytotoxicity detection and proliferation assay

Human myeloid leukemia cells K562 and NB4 purchased from American Type Culture Collection (ATCC, Rockville, MD, USA) were cultured in RPMI-1640 with 10% fetal bovine. Then effector cells (CIK or DC-CIK cells) were co-cultured with target cells (K562 or NB4 cells) at E∶T ratios of 5∶1, 10∶1, 20∶1 and 40∶1. Target cells without effector cells were used as negative control. Moreover, GT-T551 medium without any cells were used as blank control. Cell Counting Kit-8 (CCK-8) (Beyotime, Jiangsu, China) analysis was used to measure the cytotoxicity of effector cells. Meanwhile, the proliferation of both CIK and DC-CIK cells was also detected by CCK-8.

### Cytokines measure

Secreted cytokines were measured by Enzyme-linked Immunosorbent Assay (ELISA). ELISA kits for IL-35 were purchased from Abcam PLC (Cambridge, UK), and ELISA kits for IL-10 and TGF-β were purchased from R&D Systems (Minneapolis, MN, USA). Meanwhile, the two subunits of IL-35 (p35 and EBI3) and IL-35 receptor (IL-35R) (gp130 and IL-12Rβ2) mRNA were detected by Applied Biosystems 7500 Real-time PCR (Life, Grand Island, NY, USA). All primers (Table 1) were purchased from Takara Company (Takara, Dalian, China).

### Statistical analysis

The independent samples were evaluated by T test. SPSS13.0 software was used for all statistical analysis. A value of p<0.05 was considered to be statistically significant.

## Results

### DC improved the proliferative activity and cytotoxicity of CIK cells

**Figure 1:**
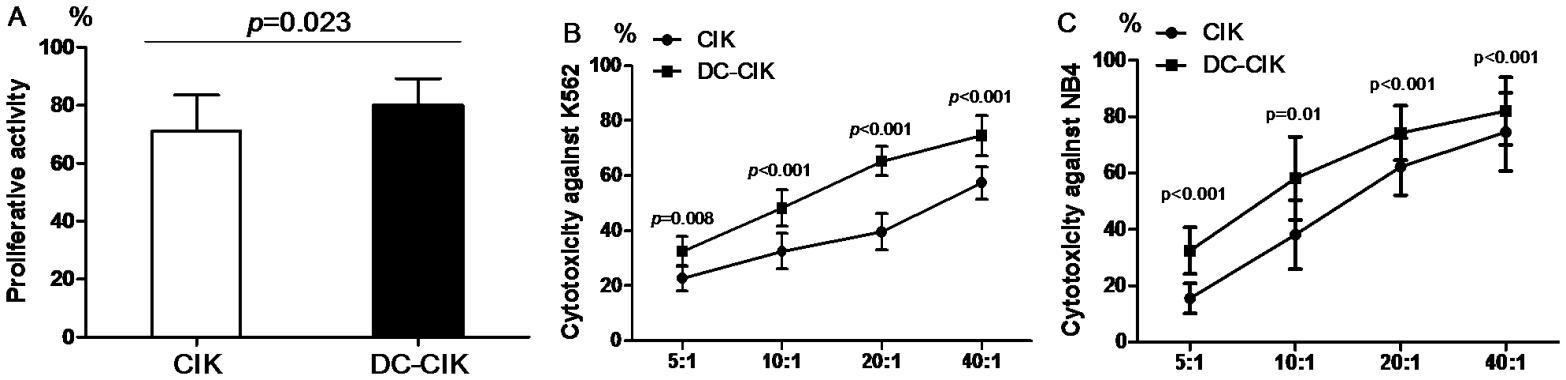
The comparison of proliferative activity and cytotoxicity between CIK and DC-CIK cells on day 28. The proliferative activity (A) and cytotoxicity against K562 cells (B) or NB4 cells (C) of DC-CIK cells were all significantly higher than those of CIK cells.

The proliferative activity and cytotoxicity of CIK and DC-CIK cells were both measured by CCK-8 on day 28. Firstly, we found the proliferation of DC-CIK cells was significant higher than that of CIK cells (p = 0.023) (Fig. 1A). Secondly, we found both CIK and DC-CIK cells had cytotoxicity against human myeloid leukemia cells K562 and NB4 cells under the ratio of 5∶1, 10∶1, 20∶1 and 40∶1, but DC-CIK cells displayed stronger anti-tumor activity than CIK cells (all p<0.01) (Fig. 1B and 1C). These data demonstrated that DC contributed to the proliferative activity and cytotoxicity of CIK cells in vitro, but the mechanism were complex. So, in order to analyze some potential mechanism related to Tregs on DC induced CIK cells, we carried out the following series of experiments.

### DC enhanced the production of NKT cells in CIK cells

**Figure 2:**
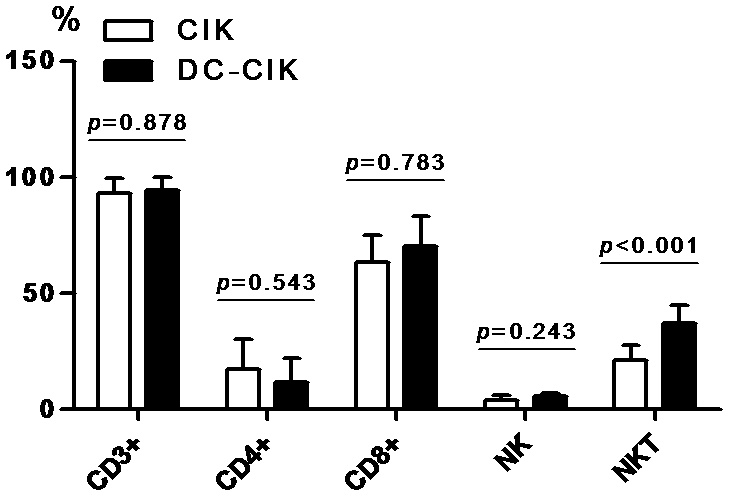
Comparison of phenotype between CIK and DC-CIK cells on day28. Compared with CIK cells, NKT cells were significantly increased in DC-CIK cells. However, there was no significant difference in the expression of CD3^+^, CD4^+^, CD8^+^ T cells and NK cells between CIK cells and DC-CIK cells.

Flow cytometry was used to analyze the phenotype of PBMC (day 0) and cultured cells on day 7, 14, 21 and 28. Compared with CIK cells, the proportion of NKT cells (CD3^+^CD56^+^, the main effector cells of CIK cells) was significantly increased in DC-CIK cells (p<0.001) (Fig. 2 shows the data of day 28). However, there was no significant difference in CD3^+^, CD4^+^, CD8^+^ T cells and NK cells between CIK and DC-CIK cells (all p>0.05) (Fig. 2 shows the data of day 28). These results indicated that application of DC during the generation of CIK cells in vitro had little effect on the expression of CD3^+^, CD4^+^, CD8^+^ T cells and NK cells, but significantly enhanced the production of NKT cells.

### DC decreased the concomitant expanded Tregs in CIK cells

**Figure 3:**
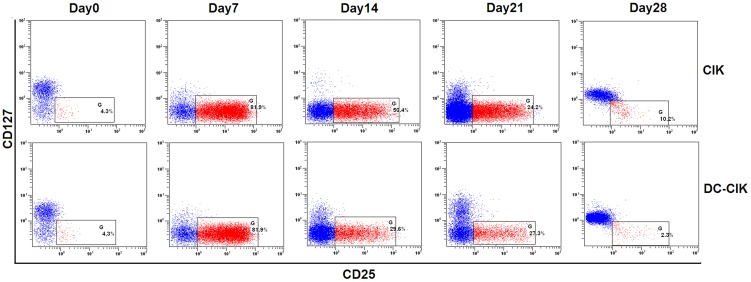
Comparison of Tregs between CIK and DC-CIK cells in a healthy adult sample. Compared with CIK cells, Tregs were decreased in DC-CIK cells throughout the process of culture.

**Figure 4:**
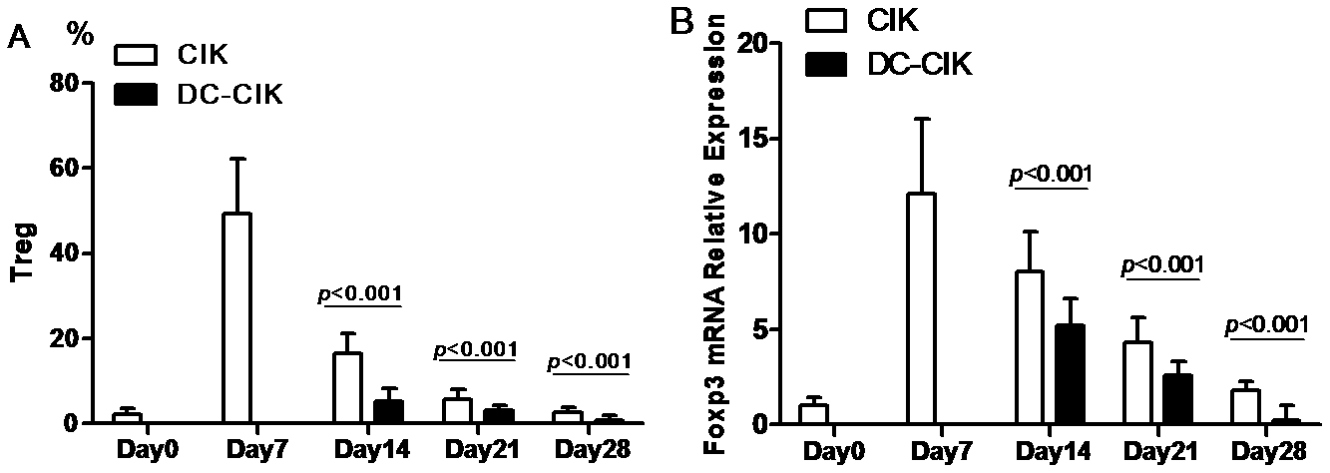
Comparison of Tregs expression between CIK and DC-CIK cells. DC significantly down-regulated the expression of Tregs in CIK cells both on cell (A) and mRNA (B) level.

In the present study, Flow cytometry was also used to analyze the expression of Tregs in PBMC (day 0) and both cultured cells on day 7, 14, 21 and 28. The data showed that non-specific TCR complexes stimulus including anti-CD3 monoclonal antibody and IL-2 significantly up-regulated the expression of Tregs in CIK cells within 28 days and the peak at day 7 for about 24 folds, but DC time-dependently decreased the concomitant Tregs in CIK cells (all p<0.001) (Fig. 3 and Fig. 4A). Moreover, we further analyzed Foxp3 mRNA expression by Real-time PCR. The data also showed that Foxp3 mRNA expression was time-dependently expanded in CIK cells and peak at day 7 for about 12 folds, but significantly decreased in DC-CIK cells (all p<0.001) (Fig. 4B).

### DC down-regulated the expression of Tregs-related IL-35 in CIK cells

Recent studies have shown that IL-10 and TGF-β could contribute to the suppressive function of Tregs in CIK cells [9], [10]. However, the newest studies showed that IL-35, rather than IL-10 or TGF-β, was required in Tregs-mediated maximal suppression [16], [19]. To further analyze the potential cytokines mechanism of Tregs in both kinds of cultured cells, we detected IL-10, TGF-β and IL-35 in the supernatant of CIK and DC-CIK cells by ELISA on day 0, 7, 14, 21 and 28 (Table 2). Analogous to the expression of Tregs, we found the amount of IL-10, TGF-β and IL-35 all rapidly increased in CIK cells within a week. Then, the IL-10 and TGF-β concentration were both significantly decreased after a maximum on day 7, but IL-35 was always increased throughout the process of culture. Meanwhile, no matter how did IL-10, TGF-β and IL-35 express in CIK cells, we found DC significantly down-regulated all of them in DC-CIK cells.

**Table 2:** The expression of IL-10, TGF-β and IL-35 in the supernatant of CIK and DC-CIK cells.

	IL-10 (pg/ml)		TGF-β (ng/ml)		IL-35(pg/ml)	
	CIK cells	DC-CIK cells	CIK cells	DC-CIK cells	CIK cells	DC-CIK cells
**Day0**	10.09±3.12	/	8.02±2.12	/	12.51±3.47	/
**Day7**	34.53±3.28#	/	43.87±4.67#	/	41.21±3.78#	/
**Day14**	29.82±2.87#	23.00±3.33^*^	38.33±3.76#	32.05±3.85^*^	46.49±5.67#	40.22±5.64^*^
**Day21**	24.62±4.26#	20.09±1.49^*^	32.08±4.86#	27.06±2.68^*^	65.08±6.43#	57.54±3.45^*^
**Day28**	22.69±2.32#	17.93±2.90^*^	28.53±3.43#	23.24±2.12^*^	74.78±6.89#	67.60±6.90^*^

# compare with day 0, p<0.05; ^*^ compare with CIK cells, p<0.05.

**Figure 5:**
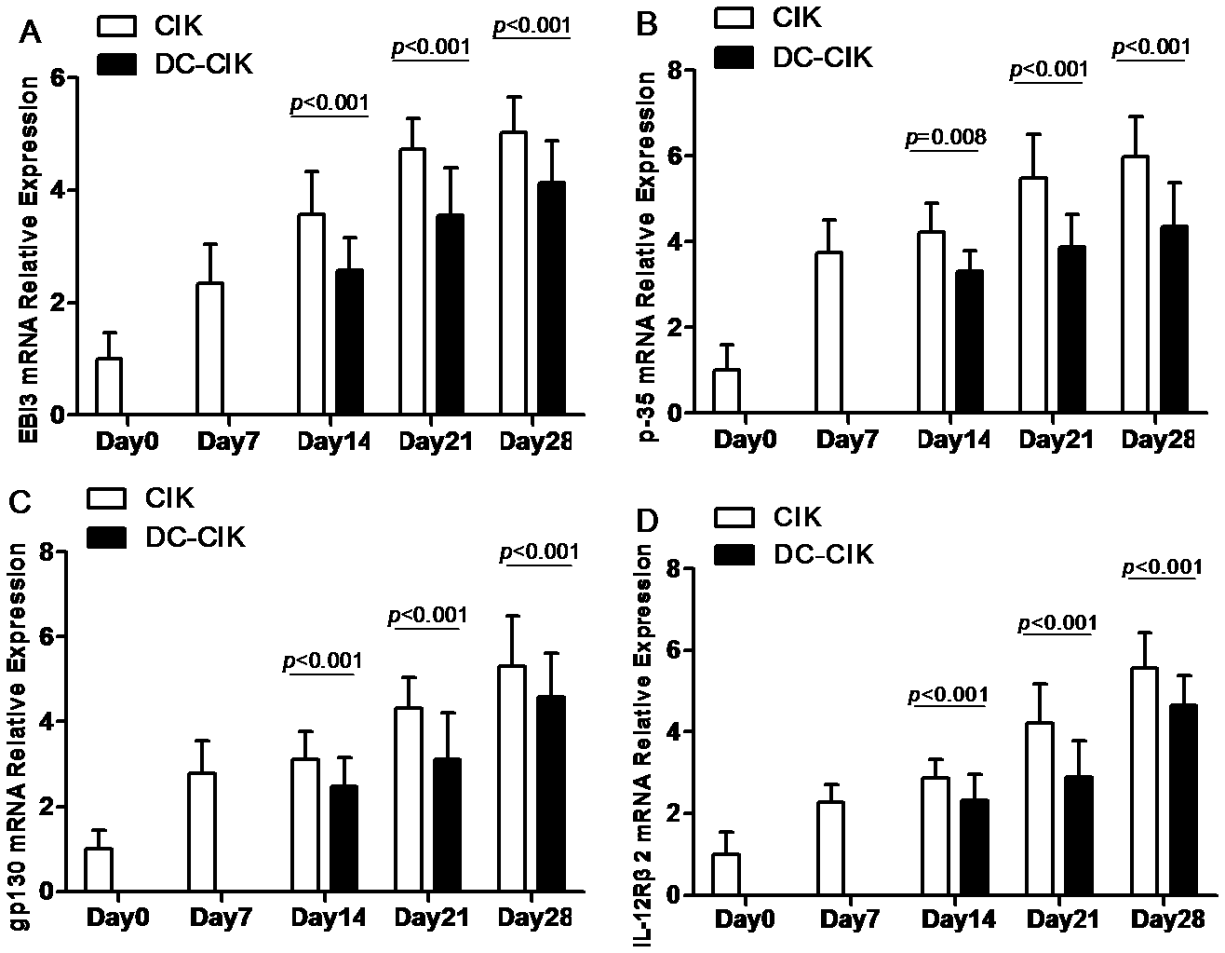
Comparison of IL-35 and IL-35R mRNA expression between CIK and DC-CIK cells. Compared with CIK cells, (A and B) EBI3 and p35 mRNA expression was significantly decreased in DC-CIK cells, (C and D) gp130 and IL-12Rβ2 mRNA expression was significantly decreased in DC-CIK cells.

In order to further investigate the activity of IL-35 in CIK and DC-CIK cells, we detected IL-35 and IL-35R expression on mRNA level by Real-time PCR. Our results showed that both in CIK and DC-CIK cells, the two subunits of IL-35 (EBI3 and p35) and two subunits of IL-35R (gp130 and IL-12Rβ2) were all increased on mRNA level compared with PBMC (day0) (all p<0.05) (Fig. 5). However, compared with CIK cells, IL-35 and IL-35R mRNA expression was significantly decreased in DC-CIK cells (all p<0.05) (Fig. 5).

## Discussion

Adoptive cell immunotherapy, as a potential new approach, holds great promises in the treatment of tumors resisted to conventional therapies [2]–[4]. Schmidt-Wolf et al. first reported that CIK cells, which are now considered as primary candidate for adoptive cell immunotherapy, had a strong anti-proliferative capacity and cytotoxicity against tumor cells [1]. It is believed that CIK cells are heterogeneous in vitro-expanded T lymphocytes with mixed NK like T cells. The anti-tumor activity of CIK cells is mostly owing to the high proliferative and cytolytic potential of CD3^+^CD56^+^ NKT cells, which increased about 100 to 1000 folds and mediated by the interaction of NKG2D receptor with MHC-unrestricted ligands on tumor cells [1], [22], [23]. In the present study, we did observed CD3^+^CD56^+^ NKT cells were significantly expanded and time-dependently increased during the generation of CIK cells within 28 days. This unique subset of non-MHC-restricted CD3^+^CD56^+^ NKT cells might effectively contribute to induce the recognition and killing of target cells. However, we also found another group of cells—Tregs were concomitantly expanded in the culture of CIK cells. Compared with PBMC, Tregs were always significantly up-regulated within 28 days in CIK cells and the peak at day 7 for about 24 folds. This abnormal phenomenon immediately caught our attention and became the focus of this study.

For the reason analysis, we known CIK cells are usually generated by the in vitro culture of mononuclear cells with IL-2 because of it is a critical lymphocyte stimulator [1]. But recent studies have noticed that IL-2 is also required for the generation and function of Tregs [24], [25]. Especially, signaling through the IL-2 receptors (IL-2R) has been shown to be critical for T cells differentiation and survival. Tregs express all three components of the high-affinity IL-2R (α: CD25, β: CD122 and δ: CD132), but effector immune cells incluing CD8^+^ T, NK and NKT cells only express incomplete two components of the low-affinity IL-2R (β: CD122 and δ: CD132) [26]. Some studies have raised the possibility that Tregs may compete with effector immune cells for IL-2 via the high-affinity IL-2R and inhibit the proliferation and function of them [27]. In the present study, because of the comparative low cumulation of IL-2 in the early stages of CIK cells culture, Tregs preferentially expanded and reached the peak within a week. But as the continuous addition and cumulation of IL-2, effector immune cells were finally triggered to be activiated, expanded and finally obtained an absolute advantage after a week. Furthemore, recent study reported that the immunosuppressive Tregs not only just concomitantly expanded in CIK cells but also had the strong biological activity. They could inhibit the anti-tumor activity of CIK cells, and TGF-β as well as IL-10 might be the important effector molecules of Tregs [9], [10]. Therefore, we believe that IL-2 may not be the optimal T cell growth factor in the culture of CIK cells, but other common γ_c_-signaling cytokines, such as IL-7, IL-15 and IL-21, may be alternative choices for the optimal culture of CIK cells.

In addition, it is believed that abrogating the effect of Tregs and Tregs-related cytokines via immunological or genetic engineering approaches will significantly improve the anti-tumor activity of CIK cells [5]. Interestingly, recent developments and advances are made in the generation of CIK cells combining with DC, and some studies specially analyzed CIK cells with or without DC to investigate Tregs feature [23], [11]–[13]. They found after co-cultured of CIK cells with DC, the CD3^+^CD56^+^ NKT cells and cytotoxicity were up-regulated, but Tregs and Tregs-related TGF-β, IL-10 were significantly down-regulated [11]–[13]. In this study, compared with conventional CIK cells, our data once again reified that DC-CIK cells exhibit stronger proliferation ability and are more effective on killing leukemia cells. Meanwhile, the expression of Tregs and TGF-β as well as IL-10 was also down-regulated in DC induced CIK cells. This results demonstrated that CIK cells co-cultured with DC could be used additionally for induction of a specific immune response at least partially via decreasing Tregs and TGF-β as well as IL-10.

Furthermore, Tregs can mediate suppression through multiple mechanisms and molecules, especially through cytokine-dependent mechanisms [14], [15]. IL-35 is a newly identified heterodimeric cytokine, containing IL-12 p35 subunit and IL-27 EBI3 subunit [16], [17]. As a novel member of IL-12 cytokine family, the source of IL-35 is different from that of other three members (IL-12, IL-23, and IL-27). The others are mainly secreted by antigen presenting cells (APCs), but IL-35 is now believed maybe specially derived from Tregs [18]. IL-35 can directly inhibit proliferation and function of effector T cells in vitro and in vivo [16], [17], and recent study showed that IL-35, rather than IL-10 or TGF-β, was required in Treg-mediated maximal suppression [16], [19]. However, whether IL-35 express in the cultured CIK cells or whether DC has an effect on its expression is unknown. Our results firstly demonstrated that IL-35 was detectable in the cultured CIK cells both in protein level and mRNA level, and might have biological activity because of IL-35R was activated at least on mRNA level. However, compared with CIK cells, DC significantly decreased the expression of IL-35 and IL-35 in DC induced CIK cells.

Moreover, some study found IL-35 could induce naive T-cells to become a novel Tregs and called them iTR35 cells [28]. These iTR35 cells had a similar suppressive function as IL-10 induced Tr1 cells and TGF-β induced iTregs did, and mediated suppression via IL-35 but not via IL-10 or TGF-β [28]. Meanwhile, iTR35 cells did not express or require the transcription factor Foxp3 and were relatively more stable than other kinds of Tregs [28]. According to the present study, we proposed that IL-35 which was initially produced by Tregs might further induce the expression of iTR35 cells in CIK cells, but these results should be regarded as preliminary and confirmed in the more extended study. We will carry out further studies focused on it in our following study.

In conclusion, we found Tregs and the novel cytokine—IL-35 time-dependently expanded during the generation of CIK cells and down-regulated their cytotoxicity, but DC siginificantly decreased the concomitant expanded Tregs as well as Tregs-related IL-35 in CIK cells and improved CIK cells-mediated cytotoxicity against leukemia cells in vitro.
